# Effects of High-Intensity Interval Training on Cognitive Function in Older Adults: A Systematic Review of Randomized Controlled Trials

**DOI:** 10.7759/cureus.114670

**Published:** 2026-08-17

**Authors:** Fatima Faisal, Andrew J Boileau

**Affiliations:** 1 Neurology, Saba University School of Medicine, The Bottom, BES; 2 Neuroscience and Neurology, Saba University School of Medicine, The Bottom, BES

**Keywords:** cognitive function, elderly, high intensity interval training, normal aging, older adults

## Abstract

High-intensity interval training (HIIT) is a form of exercise that alternates between periods of high-intensity effort and lower-intensity recovery. This review examined the effects of HIIT on cognitive function in healthy older adults. A search of the PubMed, EBSCOhost (APA PsycInfo, MEDLINE Complete, and Sociology Source Ultimate), and Epistemonikos databases was conducted using database-specific search strategies based on the concepts of high-intensity interval training (HIIT), cognition, and older adults. Eligible studies were randomized controlled trials (RCTs) involving healthy adults aged 60 years or older. Five studies met the inclusion criteria. Overall, the current evidence does not support a consistent benefit of HIIT for improving overall cognitive function in healthy older adults. However, some studies reported improvements in specific cognitive domains, including working memory and hippocampal-dependent learning, rather than generalized improvements in cognition. Interpretation of the findings was limited by heterogeneous cognitive outcome measures and the use of active exercise comparator groups, making it difficult to isolate the specific effects of HIIT. Further high-quality longitudinal RCTs are needed to determine whether HIIT provides meaningful cognitive benefits beyond conventional exercise modalities.

## Introduction and background

Previously demonstrated by exercise scientists such as Zhang and colleagues, aerobic training has advantages for the cognitive system, particularly in older adults over the age of 60 [1]. Specifically, recent systematic reviews have also demonstrated improvements in cognitive function and neuroplasticity following both aerobic and resistance exercise training [1,2]. Aerobic exercise appears to provide the greatest benefit for global cognition, whereas resistance exercise is most beneficial for executive function [1]. However, often less known, high-intensity interval training (HIIT) has the potential to include various forms of exercise (i.e., running, biking, rowing). Regardless of the workout chosen, to be classified as HIIT, a common structure is shared: shorter bouts of vigorous training followed by longer durations of recovery periods at lower intensities [3]. HIIT may incorporate resistance-based exercises, although it is not inherently a resistance-training modality. By integrating whole-body movements in an athletic manner [4], this pattern is repeated throughout the session to improve cardiovascular, muscular, and neuromuscular systems. Beyond these organ systems, emerging research suggests that HIIT may influence brain function through several physiological mechanisms, although the extent and clinical significance of these effects remain underexplored [5].

High-intensity interval training is characterized by repeated bouts of high-intensity exercise interspersed with periods of active or passive recovery. Exercise intensity is commonly prescribed using measures such as peak heart rate, heart rate reserve (HRR), or ratings of perceived exertion (RPE) [6]. This allows HIIT to be adapted across a range of exercise modalities and fitness levels [6]. A systematic review by Keating and colleagues has further demonstrated that HIIT is a safe and effective exercise modality for older adults [7]. It may produce comparable or superior improvements in cardiorespiratory fitness compared with moderate-intensity continuous training (MICT) while improving a range of health-related physical fitness outcomes [7]. Despite these physiological benefits, whether HIIT provides additional cognitive benefits beyond conventional exercise modalities remains unclear. Because HIIT is a time-efficient exercise strategy that improves cardiorespiratory fitness with a lower time commitment than traditional exercise [6], clarifying its effects on cognitive function has important implications for exercise prescription in older adults. HIIT is unique among other types of training in that it focuses on broader physiologic adaptations and helps enhance fatigue resistance [6].

Cognitive deterioration is a significant characteristic of the normal aging process. Although the world’s average life expectancy has increased over the past years, cognitive decline remains a leading feature of non-communicable diseases such as cancers, neurodegenerative diseases, strokes, and type 2 diabetes [8]. Additionally, growing evidence suggests that exercise plays an important role in protecting brain health and reducing neurodegeneration [5]. Proposed mechanisms include improvements in cerebral blood flow, cardiorespiratory fitness, neuroplasticity, and increased expression of neurotrophic factors such as brain-derived neurotrophic factor (BDNF), all of which have been associated with cognitive function [5]. Furthermore, Boa Sorte Silva et al. [9] proposed that the cognitive benefits may result from reduced losses of, or even increased, grey matter brain volume. To quantify cognitive function, researchers use screening tests such as the Montreal Cognitive Assessment (MoCA) and Mini-Mental Status Examination (MMSE). Both can be used to detect mild cognitive impairments with appropriate administration [10]. Although there are a multitude of ways to assess cognitive function, the MoCA and MMSE still remain the most common and frequently used [10].

In older adults, skills that require proficient cognitive and muscular function are crucial for continued independence and tasks of daily living. Nonetheless, gradual cognitive decline [5] and decreased muscle mass are part of the normal aging processes [11]. Consequently, older adults are an ideal population to investigate the multiple effects of HIIT. One way of implementing HIIT is by combining components of resistance training [12] and traditional continuous aerobic exercise [5] within a single workout, although HIIT can also be performed using a single exercise modality. The flexibility of HIIT allows it to be implemented using a variety of exercises depending on the training objectives. With growing interest in exercise as a strategy to preserve cognitive health during aging, the exploration of HIIT in this context becomes increasingly important. Although interest in HIIT has increased, previous systematic reviews have primarily focused on children, adolescents, and younger adults [3,13], leaving comparatively little evidence regarding the effects of HIIT on cognitive function in healthy older adults. Given the accelerated decline in both cognitive and physical function with aging, a focused synthesis of the available RCTs in this population is warranted to address this gap.

The aim of this review is to systematically examine the effects of HIIT on cognitive function in older adults by evaluating primary research, particularly RCTs. This approach will provide further insight into the relationship between exercise interventions and cognitive outcomes during normal aging. The effects of HIIT in older adults remain an area of ongoing investigation.

## Review

Methods

Search Strategy and Eligibility Criteria 

A comprehensive literature search was conducted in PubMed, EBSCOhost (APA PsycInfo, MEDLINE Complete, and Sociology Source Ultimate), and Epistemonikos in August 2026. Database-specific search strategies were developed using concepts related to high-intensity interval training (HIIT), cognition, and older adults. Medical Subject Headings (MeSH) and text word searches were used in PubMed, while equivalent keyword searches were used in EBSCOhost and Epistemonikos. The complete search strategies and database-specific filters for each database are provided in Table 1. 

**Table 1:** Search strategies and manual filters used in each database for study identification. MeSH: Medical Subject Heading

Database	Search Strategy	Filters/Limiters	Records Retrieved
PubMed	("High-Intensity Interval Training"[Mesh] OR "high intensity interval training" OR "high-intensity interval training" OR HIIT) AND ("Cognition"[Mesh] OR "cognitive function" OR cognition OR cognitive) AND ("Aged"[Mesh] OR elderly OR aged OR geriatric OR older OR "older adults" OR gerontol*)	Humans; English; Randomized Controlled Trial	58
EBSCOhost (APA PsycInfo, MEDLINE Complete, Sociology Source Ultimate)	("high intensity interval training" OR "high-intensity interval training" OR HIIT) AND ("cognitive function" OR cognition OR cognitive) AND (elderly OR aged OR geriatric OR older OR "older adults" OR gerontol*)	Human; Peer Reviewed; English	29
Epistemonikos	Entered under Advanced Search as a single string in the top main search box. ("high intensity interval training" OR "high-intensity interval training" OR HIIT) AND ("cognitive function" OR cognition OR cognitive) AND (elderly OR aged OR geriatric OR older OR "older adults" OR gerontol*)	Primary Study; Randomized Controlled Trial	50

Studies were eligible if they included healthy adults aged 60 years or older who participated in RCTs evaluating the effects of HIIT on cognitive function. For the purposes of this review, normal aging was defined as adults aged ≥60 years without diagnosed neurological, psychiatric, cardiovascular, cerebrovascular, metabolic, or other chronic medical conditions known to affect cognitive function. Studies involving disease-specific populations, mixed populations in which healthy older adults could not be analyzed separately, or participants younger than 60 years were excluded.

Study Selection and Data Extraction

**Figure 1:**
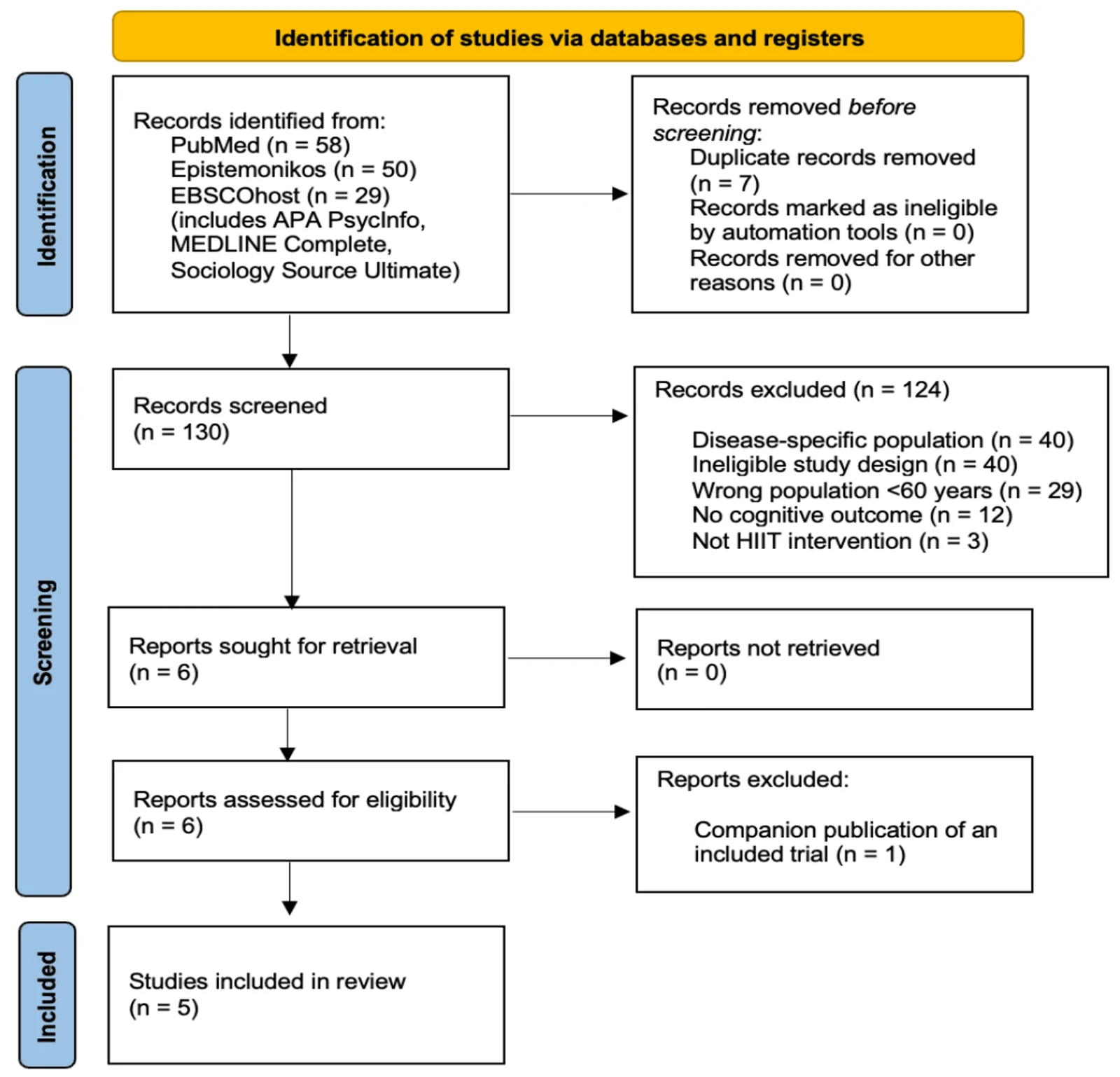
PRISMA-style flow diagram. The flow diagram was adapted from the PRISMA 2020 template [14]. Ineligible studies include review articles, study protocols, editorials, or trial registration records. PRISMA: Preferred Reporting Items for Systematic Reviews and Meta-Analyses

The Preferred Reporting Items for Systematic Reviews and Meta-Analyses (PRISMA) 2020 [14] flow diagram was used to document the study selection process (Figure 1). Duplicate records were removed manually by identifying repeated titles after alphabetically sorting the combined search results in Microsoft Excel (Microsoft Corporation, Redmond, USA). Two independent reviewers then screened the titles and abstracts, with disagreements resolved through consensus. Of the 130 records screened, a total of 124 were excluded because of disease-specific populations (n = 40), ineligible study designs (review articles, study protocols, editorials, or trial registration records) (n = 40), participants younger than 60 years (n = 29), absence of cognitive outcomes (n = 12), or interventions that were not HIIT (n = 3). The full texts of six studies were assessed for eligibility. One companion publication was excluded to avoid double-counting data from the same participants, leaving five studies for inclusion in this review. A formal meta-analysis was not performed because of substantial methodological and outcome heterogeneity among the included studies. Instead, a narrative synthesis was conducted. Studies were compared according to participant characteristics, HIIT protocol, comparator intervention, intervention duration, cognitive outcome measures, and overall direction of findings to identify similarities and differences across the included trials.

Risk of Bias Assessment

The Cochrane Risk of Bias 2 (RoB 2) tool [15] was selected to assess the methodological quality of the included RCTs. Five domains were evaluated: bias arising from the randomization process, bias due to deviations from intended interventions, bias due to missing outcome data, bias in measurement of the outcome, and bias in selection of the reported result. Each study was assigned an overall judgment for risk of bias (Table 4). These included low risk of bias, some concerns, or high risk in accordance with the RoB 2 algorithm. Disagreements between reviewers were resolved through consensus.

Results

Of the five RCTs evaluated, three studies included inactive/non-exercising sedentary older adults (Kwok et al. 2024; Okamoto et al. 2019; Simonsson et al. 2023) [16-18], with one of them enrolling women exclusively [16]. The remaining two studies examined generally healthy and normally aged older adults (Table 2). Furthermore, two out of the five studies used the MoCA to assess cognitive function. The evidence regarding the effects of HIIT on cognition is summarized in Table 3.

**Table 2:** Study design characteristics. MoCA: Montreal Cognitive Assessment [10]

Sample of article's results/characteristics	# of studies
Randomized controlled trial study design	5
MoCA for cognitive function measurement	2
Head-to-head comparison	5
Inactive, non-exercising, sedentary population	3
Female-only older adults	1

**Table 3:** Evidence table. Note differences between studies in interval training vs continuous training for MIT [18,20]. IWT: interval walking training, NWT: normal walking training, TMT-A: trail-making test A, WG: within-group, BG: between-group, HIIT: high-intensity interval training, HR: heart rate, MICT: moderate-intensity continuous training, MoCA: Montreal Cognitive Assessment [10], HIT: high-intensity training, MIT: moderate intensity training, EPMCS: episodic memory composite score, VSA: visuospatial ability, PS: processing speed, EF: executive function, AHIIT-DWR: aquatic high-intensity interval training-deep water running, HRR: heart rate reserve, LHIIT: land-based high-intensity interval training, MMSE: Mini-Mental Status Examination [10], LIT, low-intensity training; PALTEA: paired associates learning total errors adjusted

First Author and Year	Level of Evidence and Study Design	Study Population	Therapy/Exposure	Outcome/Results	Key numerical data
Okamoto, T. [17], 2019	Level 1 RCT	Non-regular exercising older adults n=68	1) IWT: 70% and 40% peak aerobic capacity for intervals. 2) NWT: 50% peak aerobic capacity, 8000 steps/day.	No significant differences observed between IWT and NWT.	Time for TMT-A: WG improvement in both IWT (p<0.01, d=0.86) and NWT (p<0.01, d=1.23). Mean ± SE graphically reported. No significant BG effect reported.
Zotcheva, E. [19], 2022	Level 1 RCT	Older adults n=945	1) HIIT: 4 min. at 85–95% of peak HR with 3 min. active breaks (60–70% of peak HR), 2) MICT: continuous training at 70% of peak HR. 3) Control: (unsupervised)	No significant differences between HIIT and MICT.	HIIT MoCA scores compared to control: adjusted B 0.32, 95% CI − 0.22, 0.85. MICT MoCA scores compared to control: adjusted B 0.21, 95% CI − 0.32, 0.73.
Simonsson, E. [18], 2023	Level 1 RCT	Non-exercising older adults n=68	1) Supramaximal HIT: 60% of maximum mean power output, progressing intensity throughout 10×6 sec. intervals with 54 sec. recovery periods. 2) MIT: 40% of maximum aerobic power for 3×8 min. intervals with 3 min. recovery periods.	No significant differences were observed between HIT and MIT in global cognition.	Global cognitive composite score between HIT and MIT (mean=0.11, 95% CI [−0.03, 0.24]). No significant change in EPMCS, VSA, PS, EF.
Kwok, M.M.G. [16], 2024	Level 1 RCT	Inactive women aged 60+ n=70	1) AHIIT-DWR: DWR (2 min. bouts at 80% HRR) alternating with active recovery bouts (1 min. at 60% HRR). 2) LHIIT: treadmill running at the same intensity.	No significant group differences were observed between AHIIT-DWR and LIIT.	MMSE: No significant between-group difference (p>0.05, ES=0.00). MoCA: Significant time effect (p<0.01, ES=0.22); no significant group effect.
Blackmore, D.G. [20], 2024	Level 1 RCT	Healthy aged individuals n=151	1) HIIT: 4 min. running intervals at 85-95% peak HR with 3 min. recovery. 2) MIT: continuous treadmill walking at 60-75% peak HR. 3) LIT: balance, range of motion activities at 45-55% peak HR.	Preserved hippocampal volume and PALTEA learning performance (fewer errors) improved for HIIT and remained up to 5 years.	PALTEA learning performance at 6 months; effects maintained up to >48 months; LIT:HIIT ES=0.72, MIT:HIIT ES=0.74. Mean ± SE graphically reported; two-way RM-ANOVA (time × exercise intensity effect; F (10,738) = 2.272, p = 0.012).

Risk of Bias Assessment 

**Figure 2:**
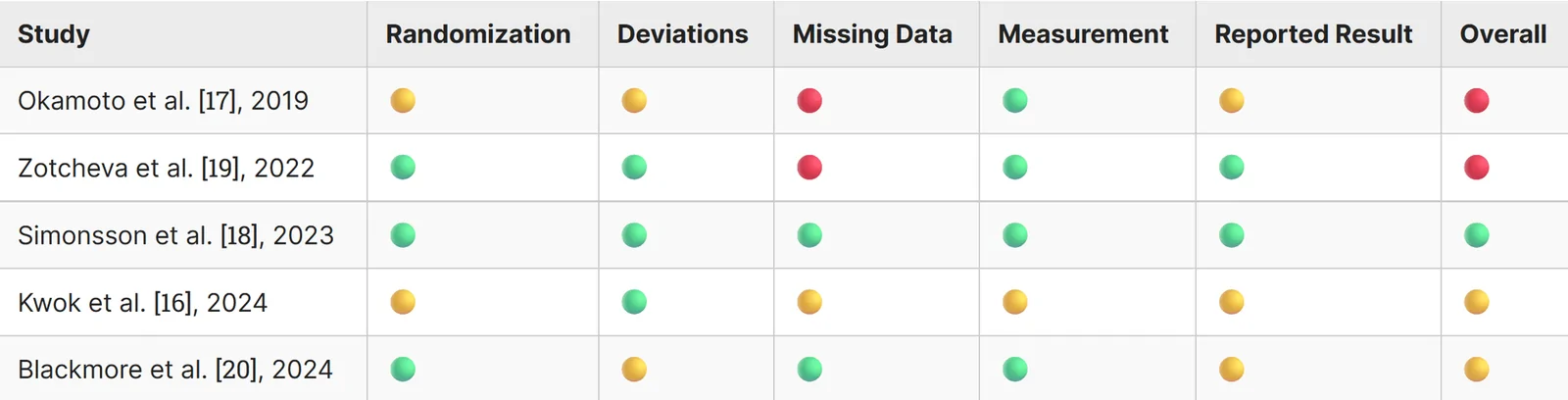
RoB 2 traffic light plot. RoB 2: Cochrane Risk of Bias 2; Yellow: some concern; Green: low risk of bias; Red: high risk of bias

The risk of bias assessment for the five included RCTs is summarized in Table 4 and Figure 2. One study was judged to be at low risk of bias, two studies were judged as having some concerns, and two studies were judged to be at high risk of bias. High risk of bias was primarily attributable to bias arising from missing outcome data. Studies judged as having some concerns most commonly reflected limitations in the reporting of the randomization process, deviations from intended interventions, or selection of the reported results.

**Table 4:** RoB 2 risk of bias assessment. RoB 2: Cochrane Risk of Bias 2 [15].

Study	Randomization Process	Deviation from Intended Intervention	Missing Outcome Data	Measurement of the Outcome	Selection of the Reported Result	Overall Risk of Bias
Okamoto et al. [17], 2019	Some concerns	Some concerns	High	Low	Some concerns	High
Zotcheva et al. [19], 2022	Low	Low	High	Low	Low	High
Simonsson et al. [18], 2023	Low	Low	Low	Low	Low	Low
Kwok et al. [16], 2024	Some concerns	Low	Some concerns	Some concerns	Some concerns	Some concerns
Blackmore et al. [20], 2024	Low	Some concerns	Low	Low	Some concerns	Some concerns

Okamoto et al. [17] randomized 68 sedentary, community-dwelling older adults (mean age 70 ± 4 years) to interval walking training (IWT) or normal walking training (NWT). Adherence was 74% in the IWT group and 79% in the NWT group. IWT alternated between fast and slow intervals, which categorized it as a form of HIIT. Participants assigned to NWT completed 8000 steps/day at 50% peak aerobic capacity at least three times a week. IWT consisted of five three-minute fast intervals at 70% of aerobic capacity or higher, and alternated with slow intervals at 40% of peak aerobic capacity for at least 3 days a week. Effects of these interventions were observed using the trail-making test-A and B (TMT-A, TMT-B), with a shorter time indicating higher cognitive function. After the 20-week study period, decreased time for both TMT-A and TMT-B (p<0.05) was observed in both IWT and NWT when compared to baseline. Mean ± SE were graphically reported. Significant between-group differences were not observed for either TMT-A or TMT-B. Secondarily, significant decreases in arterial stiffness measured by carotid-femoral pulse wave velocity (cfPWV) were observed in both groups after the exercise intervention (p<0.05). IWT resulted in a greater reduction in arterial stiffness than NWT (p=0.03).

Zotcheva et al. [19] conducted an RCT in 945 older adults with a mean age of 78.2 ± 2.02 (mean ± SD) by the end of the study. The effects of HIIT on global cognition and mild cognitive impairment (MCI) were assessed using the Norwegian version of the Montreal Cognitive Assessment (MoCA) at the 5-year follow-up. Participants were randomized in a 2:1:1 ratio between HIIT, moderate-intensity continuous training (MICT), and a control group. After 5 years, adherence to MICT was 75%, HIIT was 76%, and the control group was 96%. For a total of 40 minutes, HIIT consisted of 4-minute working periods at 85-95% of peak heart rate with 3-minute active breaks (60-70% of peak heart rate). MICT consisted of continuous training for a total of 50 minutes at a moderate intensity (70% of peak heart rate). The unsupervised control group was advised to follow the 2012 national recommendations for physical activity (i.e., 30 minutes of moderate-intensity physical activity almost daily, or two weekly sessions of aerobic exercise over 5 years). The authors found that combined HIIT and MICT resulted in slightly higher MoCA scores (adjusted B 0.26, 95% CI − 0.17, 0.69) and lower odds of MCI (adjusted OR 0.86, 95% CI 0.66, 1.13), though these results did not reach statistical significance. When analyzed separately, HIIT (adjusted B = 0.32, 95% CI −0.22 to 0.85; adjusted OR = 0.81, 95% CI 0.57-1.15) and MICT (adjusted B = 0.21, 95% CI −0.32 to 0.73; adjusted OR = 0.92, 95% CI 0.66-1.27) showed similar trends compared with the control group, although none of the confidence intervals excluded the null value. These results were also not statistically significant.

Simonsson et al. [18] examined 68 non-exercising older adults aged 65 years and older following three months of an adapted supramaximal high-intensity interval training (HIT) or moderate-intensity training (MIT). Subjects had not engaged in regular physical activity at moderate to high intensity over the past year, regardless of previous exercise experience. Training was computer-guided on indoor cycling bikes consisting of 10 six-second intervals with 54 seconds of recovery for a total of 20 minutes biweekly. For supramaximal HIT, intensity progressed, starting from 60% of maximum mean power output. For the MIT group, three 8-minute intervals with 3-minute recovery periods for a total of 40 minutes at 40% of maximum aerobic power were performed. The primary outcome was global cognitive function with secondary domain-specific cognitive functions. There were no significant differences observed in the global cognition composite score between supramaximal HIT and MIT (mean=0.11, 95% CI [−0.03, 0.24]) (p=0.940, ES=0.019). Statistically significant between-group differences for supramaximal HIT were observed for the working memory composite score (mean=0.32, 95% CI [0.01, 0.64]) (p=0.052, ES=0.500) as a secondary outcome. In general, independent of grouping, there were no statistical changes in episodic memory composite score, visuospatial ability, processing speed, and executive function [18].

Kwok et al. [16] investigated the effect of HIIT on 70 inactive women aged 60 and older. The primary outcome measured was cognitive function measured through MMSE and MoCA assessments. Participants were randomized into two groups and performed a total of 16 30-minute sessions 2x/week. AHIIT-DWR (aquatic high-intensity interval training-deep water running) consisted of 2-minute bouts of DWR at 80% heart rate reserve (HRR) alternating with active recovery bouts for 1 minute at 60% HRR, for 10 rounds. LHIIT (land-based high-intensity interval training) consisted of treadmill running bouts at 80% HRR, alternating with active recovery bouts for 1 minute at 50% HRR for 10 rounds. Cognitive function was assessed before and after the 8-week intervention period. Comparison between AHIIT-DWR and LHIIT was assessed with MMSE and MoCA. Time effect, group effect, and group-time effect showed no significant differences except a time effect in MoCA (p<0.01, ES=0.22) [16]. The authors made no inference from that finding. 

Blackmore et al. [20] evaluated the long-term effects of high-intensity interval training (HIIT), moderate-intensity training (MIT), and low-intensity training (LIT) in 151 healthy older adults aged 65-85 years over a 5-year follow-up. Cognitive function was assessed using the Paired Associates Learning total errors adjusted (PALTEA) score from the Cambridge Automated Neuropsychological Test Automated Battery (CANTAB), and hippocampal volume was also evaluated. HIIT consisted of 4-minute running intervals at 85-95% of peak heart rate (HR), followed by 3-minute recovery periods. MIT consisted of continuous treadmill walking at 60-75% of peak HR, while LIT consisted of a mix of stretching, balance, and range of motion activities at 45-55% peak HR. Participants assigned to HIIT demonstrated significantly greater improvements in PALTEA performance than both LIT and MIT (time × exercise intensity interaction: F (10,738) = 2.272, p = 0.012). Post hoc analyses confirmed superior performance following HIIT compared with LIT (p = 0.004, ES = 0.72) and MIT (p = 0.008, ES = 0.74). Improvements in learning performance were maintained for up to 5 years, with the greatest gains occurring during the first 6 months. Additionally, hippocampal volume was preserved in the HIIT group, whereas significant reductions were observed in both the LIT (p < 0.001) and MIT (p < 0.0001) groups. Mean ± SD graphically reported.

Discussion

This systematic review synthesized evidence from RCTs investigating the effects of HIIT on cognitive function in healthy older adults. Taken together, the included studies demonstrated inconsistent evidence regarding the cognitive benefits of HIIT in healthy older adults. Interpretation of the findings should consider the methodological quality of the included studies. Four of the five RCTs were judged to have either some concerns or a high risk of bias. The primary source of high risk of bias was missing outcome data resulting from participant attrition, particularly in studies with longer follow-up periods. Consequently, although several studies demonstrated favorable cognitive outcomes following HIIT, these findings should be interpreted with appropriate caution.

Overall, four of the five included studies found no significant between-group improvements in overall cognitive function following HIIT compared with their respective comparator interventions. However, two studies reported improvements in specific cognitive domains. Simonsson et al. [18] observed a between-group effect favoring HIIT for working memory, while Blackmore et al. [20] reported sustained improvements in hippocampal-dependent learning and preserved hippocampal volume following HIIT. Across the included studies, substantial heterogeneity was observed in intervention protocols, comparator groups, cognitive outcome measures, and follow-up duration, precluding direct comparison of results.

Common limitations

Through the review of the five RCTs presented, common limitations and strengths helped provide insight into possible avenues of research. One major limitation across all studies includes the inconsistent measurement parameters for cognitive function and decline. Only two of the five studies used the standard MoCA (Kwok et al. [16]; Zotcheva et al. [19]). The remaining studies assessed relevant aspects of cognition; however, differences in the cognitive domains evaluated and scoring methods limited direct comparisons across studies (Okamoto et al. [17]; Simonsson et al. [18]; Blackmore et al. [20]). This leaves an ambiguous comparison of the true effect of HIIT and may require a refined methodological process in novel RCTs moving forward. Another common limitation among the reviewed studies related to the overall intervention duration being generally insufficient to produce detectable cognitive function changes (Okamoto et al. [17]; Simonsson et al. [18]; Blackmore et al. [20]). Therefore, it was difficult to extract the effects of how long cognitive decline could be protected for. The lack of a non-exercising control group also led to a potential Hawthorne effect (Kwok et al. [16]; Okamoto et al. [17]; Simonsson et al. [18]; Blackmore et al. [20]), since it was unclear if the results were from the exercise intervention itself. Future studies should aim to build upon the current knowledge of the effects of HIIT on cognitive function in older adults. Despite these limitations, several important observations emerged from the included studies.

Study-Specific Discussion: Strengths and Limitations 

Okamoto et al. [17] found no significant between-group differences in cognitive function despite greater reductions in arterial stiffness following interval walking training (IWT). This suggests that vascular adaptations may precede measurable cognitive improvements, particularly over a relatively short intervention period. Reduced arterial stiffness may preserve cerebral microvascular function and limit age-related changes in frontal and subcortical brain regions associated with cognitive decline [21,22]. Future longitudinal studies are needed to determine whether these vascular improvements translate into long-term cognitive benefits.

A strength of this study was successful randomization, with no significant baseline differences between groups. Although the IWT group expended less energy and completed fewer steps than the normal walking group, it achieved greater improvements in arterial stiffness, suggesting that exercise intensity may be more important than exercise volume for promoting vascular health. This finding is consistent with Brown et al., who reported that exercise intensity may have a stronger association with cognitive function than the quantity of physical activity [23]. However, the absence of significant between-group cognitive differences indicates that the cognitive benefits of HIIT remain uncertain. Limitations included a relatively small sample size, low adherence, and participant attrition, reducing statistical power and increasing the risk of a Type II error. Larger studies with longer follow-up periods are needed to determine whether improvements in vascular function are accompanied by measurable cognitive benefits [17].

Zotcheva et al. [19] found that neither HIIT nor MICT was associated with significant improvements in global cognitive function or reduced odds of mild cognitive impairment (MCI) compared with the control group after 5 years. Interestingly, stratified analyses suggested that exercise was associated with higher global cognition and lower odds of MCI in men, but not women [19]. The authors proposed that the prescribed exercise intensity may not have been sufficient to produce measurable between-group differences, while participant attrition over the lengthy follow-up may have reduced variability between groups. These findings suggest that long-term participation in HIIT alone may not be sufficient to improve overall cognitive function and that factors such as sex and individual characteristics may influence the cognitive response to exercise.

A major strength of this study was its large sample size and extended 5-year intervention period, providing valuable insight into the long-term effects of exercise on cognition. However, the absence of baseline MoCA assessments limited the ability to determine whether participants had subtle cognitive impairment before enrollment. Moreover, if participants were already regular exercisers, the non-significant results could have been explained by a potential ceiling effect, with additional HIIT acting marginally to their current exercise regimens. Additionally, volunteer bias may have reduced the generalizability of the findings. Since adherence was measured using a questionnaire validated in young men [24], recall bias may also have been present [19]. These findings suggest that HIIT alone was not superior to MICT for preserving global cognitive function in this cohort, highlighting the complexity of cognitive aging and the likelihood that multiple factors influence cognitive health. Future studies should further investigate potential sex differences and identify factors contributing to the variability in cognitive responses to HIIT among older adults [19].

Simonsson et al. [18] found that supramaximal HIT did not improve overall cognitive function compared with MIT. However, a moderate effect size favoring supramaximal HIT was observed for working memory. Although this finding did not reach conventional statistical significance (p = 0.052), the accompanying confidence interval and moderate effect size suggest a potential domain-specific benefit that warrants further investigation. The authors proposed that the greater improvements in aerobic fitness (VO₂peak) and muscular strength achieved with HIIT may have contributed to these findings. Often combined in HIIT, both aerobic and resistance training have similar and unique physiological effects that positively influence working memory [25]. Future studies should determine whether these domain-specific benefits are reproducible over longer intervention periods and identify factors associated with individual responsiveness to HIIT.

Strengths of this study included appropriate randomization, blinding procedures, and standardized progression of exercise intensity. However, the comparator group also performed moderate-intensity interval training, making it difficult to distinguish whether the observed cognitive changes were attributable specifically to HIIT or to interval exercise more generally. In addition, some participants were more physically active at baseline than intended, which may have reduced the contrast between intervention groups. Simonsson et al. [18] also assessed cardiorespiratory fitness (V̇O₂peak) as a secondary outcome. Baseline V̇O₂peak values for the study population were lower than those reported in similar studies of older adults [26], suggesting that the recruited participants were still relatively inactive despite some participants exceeding the intended activity criteria. Moreover, the use of V̇O₂peak rather than V̇O₂max may have underestimated participants' true aerobic capacity because some individuals may not have achieved maximal oxygen uptake during testing. Although a sensitivity analysis using age-adjusted V̇O₂max criteria did not meaningfully alter the findings, there was a trend favoring V̇O₂max. Future studies should obtain direct V̇O₂max measurements where feasible to improve the precision of exercise prescription and fitness assessment [18].

Kwok et al. [16] found no significant between-group differences in cognitive function between aquatic high-intensity interval training-deep water running (AHIIT-DWR) and LHIIT as measured by the MMSE and MoCA. Instead, the authors suggest that the within-group differences may be explained by an increase in cerebral blood flow and cardiovascular function, which may indirectly contribute to the functioning of neurotransmitters and cognition of the brain [27]. However, these mechanisms were not directly measured. Consequently, the findings suggest that exercise modality may have little influence on cognitive outcomes when exercise intensity is comparable. From a clinical perspective, aquatic HIIT may represent a practical alternative for older adults who are unable to tolerate weight-bearing exercise. In particular, for postmenopausal women at increased risk of osteoporosis, AHIIT-DWR may provide a feasible, lower weight-bearing alternative to land-based HIIT while producing comparable cognitive outcomes.

Notable strengths of this study include adherence to the exercise intervention and matching participant exercise intensity between both groups using HRR. Very few studies have looked at AHIIT-DWR and LHIIT at matched exercise intensities, which further supports AHIIT-DWR being a suitable alternative when weight-bearing exercise is a limiting factor. The generalizability of the findings in this study is limited by the participation of only women. Future longitudinal studies that have a larger sample size and include mixed sexes may be warranted [16].

Unlike the other studies included in this review, Blackmore et al. [20] was the only study to demonstrate sustained improvements in cognitive performance following HIIT. Improvements in PALTEA performance were maintained for up to 5 years, indicating that HIIT may have sustained effects on hippocampal-dependent learning and memory. In comparison to MIT or LIT, higher exercise intensity may produce more durable cognitive benefits. Preservation of hippocampal volume following HIIT may partly explain these findings, as hippocampal integrity is closely associated with learning and memory [20]. However, improvements were limited to hippocampal-dependent cognition and were not observed across other cognitive domains, suggesting that HIIT may preferentially benefit specific aspects of cognition rather than overall cognitive function. The long-term maintenance of cognitive benefits following only 6 months of HIIT may have important clinical implications for older adults, for whom sustained participation in high-intensity exercise may become increasingly challenging with age. However, as this was the only study in the present review to demonstrate long-term cognitive improvements, additional RCTs are needed to confirm whether these benefits are reproducible in broader older adult populations.

Strengths of this study included appropriate randomization, an extended follow-up period, and objective assessment of hippocampal volume using high-resolution 7-Tesla MRI. However, the exclusion of individuals with comorbidities may limit the generalizability of the findings to the broader older adult population. In addition, because the intervention consisted of treadmill-based HIIT, it remains unclear whether similar cognitive benefits would be observed with other HIIT modalities, such as cycling or swimming [20].

Baseline Activity as a Possible Source of Heterogeneity

Analysis of the five included studies identified baseline participant characteristics, including physical activity status, as a potential source of heterogeneity. Three studies specifically recruited inactive or non-exercising older adults [16-18], whereas the remaining studies enrolled community-dwelling older adults without requiring baseline inactivity [19,20]. Although Blackmore et al. [20] reported improvements in hippocampal-dependent learning, whereas the studies recruiting inactive participants did not demonstrate significant between-group improvements in overall cognitive function [16-18], these observations should be interpreted cautiously because of the small number of studies and substantial methodological heterogeneity. Consequently, whether baseline physical activity or fitness influences the cognitive response to HIIT remains unclear. Participants with higher baseline fitness may tolerate repeated high-intensity exercise more effectively than previously inactive individuals, who may require an initial adaptation period before achieving a sufficient training stimulus. However, this hypothesis could not be evaluated within the present review because baseline fitness was not directly examined as an effect modifier. Future RCTs should investigate whether baseline physical activity or fitness modifies the cognitive response to HIIT and whether an initial adaptation period improves outcomes in previously inactive older adults.

Potential Role of Interval Training

One possible explanation for the observed findings is that the interval structure of HIIT, rather than exercise intensity alone, may contribute to the cognitive benefits associated with exercise. Although Blackmore et al. [20] demonstrated greater cognitive benefits with HIIT than continuous moderate- and low-intensity exercise, Zotcheva et al. [19] found no differences between HIIT and MICT. However, Simonsson et al. [18] reported similar cognitive outcomes following high- and moderate-intensity interval training. Collectively, these findings raise the possibility that interval structure, rather than exercise intensity alone, may contribute to the cognitive effects observed. However, none of the included studies directly compared interval and continuous exercise performed at the same high intensity. Therefore, the independent contribution of interval training cannot be determined from the available evidence. RCTs directly comparing high-intensity interval and high-intensity continuous exercise using standardized cognitive outcome measures are needed to determine whether the interval component independently contributes to cognitive benefits.

Based on the current literature, the lack of consistent results on cognitive function and decline following HIIT should prompt future research to circumvent this uncertainty. In particular, controlling for all variables with enough power in an ANOVA table stratified by activity, sex, age groups, baseline cognitive function, and monitored for as long as available is warranted.

## Conclusions

From the reviewed studies, it appears that current evidence does not support a consistent benefit of HIIT for overall cognitive function in healthy older adults. Most included studies reported no significant between-group improvements in global cognitive outcomes compared with control interventions. However, improvements in specific cognitive domains, particularly working memory and hippocampal-dependent learning, were observed in some studies, suggesting that HIIT may preferentially influence selected aspects of cognition rather than global cognitive performance. These findings highlight the need for additional high-quality RCTs to clarify the mechanisms underlying these domain-specific effects, identify the populations most likely to benefit, and determine the optimal HIIT prescription. Further research comparing HIIT with other exercise modalities is also warranted to establish whether HIIT provides meaningful cognitive and practical advantages for older adults. Such evidence may help guide exercise recommendations aimed at preserving cognitive health while addressing the needs and capabilities of an aging population.
